# Can your eyes taste freshness? The role of visual realism, motion cues, and spatial imagery ability in juice packaging

**DOI:** 10.3389/fpsyg.2026.1826220

**Published:** 2026-05-28

**Authors:** Yang Zhang, Zichen Lyu, Lin Yang

**Affiliations:** 1 Department of Design, Graduate School, Hanyang University, Seoul, Republic of Korea; 2 Academy of Arts & Design, Tsinghua University, Beijing, China; 3 Department of Design, Graduate School, Chung-Ang University, Seoul, Republic of Korea

**Keywords:** image realism, implied motion cues, juice packaging, perceived food freshness, perceived image dynamism, spatial imagery ability

## Abstract

**Purpose:**

This study examines how visual realism and implied motion cues in juice packaging shape consumers’ perceived food freshness and purchase intention in sensory-constrained evaluation contexts. It further investigates perceived image dynamism as a mediating mechanism and spatial imagery ability as an individual-difference boundary condition.

**Design/methodology/approach:**

Study 1 tested the effect of image realism (realistic vs. stylized) on perceived freshness, healthfulness, and purchase intention. Study 2 manipulated implied motion cues while holding style constant and examined mediation by perceived image dynamism. Study 3 tested the moderating effect of spatial imagery ability. Data were collected via online surveys and analyzed using ANOVA and PROCESS-based mediation and moderated-mediation models.

**Findings:**

Realistic imagery increased perceived food freshness, perceived healthfulness, and purchase intention. Implied motion cues enhanced perceived food freshness by increasing perceived image dynamism. Spatial imagery ability moderated this mechanism: the effect of perceived image dynamism on perceived food freshness was stronger among consumers with lower spatial imagery ability, suggesting that these consumers relied more heavily on externally presented motion cues when forming freshness judgments.

**Originality**/**value:**

This research advances the juice packaging and sensory marketing literature by integrating visual realism, implied motion cues, perceived image dynamism, and spatial imagery ability into a unified framework. The findings show that packaging imagery functions not merely as decoration but as an informational cue through which consumers infer product freshness. The study also highlights the role of consumer cognitive differences in shaping the effectiveness of visual packaging cues.

## 1 Introduction

Freshness is widely recognized as one of the most influential criteria in food choice, often exceeding price or convenience in importance (Ragaert et al., 2004). As consumers increasingly rely on prepackaged foods and online food purchases, those with limited time and constrained cognitive resources depend more heavily on external cues to make rapid judgments about food quality (Gil-Pérez et al., 2020). In this process, packaging serves not only protective and informational functions but also communicative ones, attracting attention, shaping first impressions, and influencing purchase decisions (Krishna et al., 2017). As the first point of contact between consumers and food products, food packaging can rapidly evoke cognitive and affective responses through visual elements such as color, imagery, and shape, thereby influencing perceptions of flavor, freshness, and quality (Machiels and Karnal, 2016).

Among these visual elements, package imagery is particularly important. Food packages vary substantially in how products are visually represented, ranging from highly realistic photographs to stylized illustrations or abstract graphics. Prior research suggests that realistic imagery reduces consumers’ psychological distance from a product and enhances evaluation outcomes (Ketron et al., 2021). Consistent with construal level theory (CLT; Trope and Liberman, 2010), concrete and vivid depictions tend to increase feelings of immediacy and presence, whereas abstract depictions may create a more symbolic or distant impression. In food contexts, realistic fruit imagery is more likely to evoke associations with naturalness, freshness, and high quality, thereby fostering more favorable judgments of product attributes (Becker et al., 2011). Accordingly, image style represents an important packaging cue that may shape perceived food freshness.

Beyond image style, implied motion cues embedded in packaging imagery constitute another common visual strategy. Many juice packages rely on static visuals augmented by motion-related cues, such as splashing, pouring, spraying, or bursting, to evoke a sense of movement (Gvili et al., 2015; Yu et al., 2022). Although such cues do not involve actual movement, they may make the image appear more fluid, vivid, and immediate, thereby influencing consumers’ inferences about product state. Existing studies indicate that food images containing implied motion can increase attention, perceived healthfulness, expected taste, and product attractiveness (Elder and Krishna, 2022; Xiong et al., 2023). However, in the context of food packaging, there is still limited direct evidence as to whether such cues enhance freshness judgments by increasing consumers’ subjective sense of image dynamism.

Not all consumers, however, respond equally to visual packaging cues. Mental simulation is often invoked as a key explanatory mechanism through which visual cues influence judgment, yet its strength may depend on individual differences in cognitive ability (Pearson, 2019). One particularly relevant construct is spatial imagery ability, defined as the capacity to visualize and manipulate three-dimensional images mentally (Zacks, 2008). Consumers with higher spatial imagery ability may be better able to generate internal representations of product states from limited visual information, whereas those with lower spatial imagery ability may rely more heavily on explicit visual cues provided by packaging. However, the role of spatial imagery ability in juice packaging contexts remains underexplored. It therefore merits examination as a potential boundary condition in the process linking visual cues, perceived image dynamism, and freshness judgment.

Although prior studies have separately examined image realism, implied motion, and consumer perception, the literature remains incomplete in several respects. First, most existing work considers image realism and implied motion in isolation, with limited effort to integrate these visual cues into a unified framework of consumer perception; as a result, it remains unclear how they jointly shape inferences about food freshness. Second, although mental simulation is frequently used to explain why visual cues affect consumer perception, it is often treated as a *post hoc* interpretation rather than being systematically tested as a mediating mechanism in the formation of freshness judgments. Third, individual cognitive differences, especially spatial imagery ability, remain underexplored in packaging contexts, despite evidence that they influence how people process visual and motion-related information. Accordingly, a theoretical framework that integrates visual cue characteristics with cognitive processes is essential for a more complete understanding of perceived food freshness.

Against this background, the present research develops a framework that incorporates main effects, underlying mechanisms, and boundary conditions. Specifically, image realism and implied motion are conceptualized as external visual cues in packaging that strengthen freshness-related inferences by enhancing consumers’ perceived image dynamism and mental simulation. At the same time, spatial imagery ability is treated as an individual-difference variable that may influence the depth and efficiency of mental simulation, thereby moderating the effects of visual cues on freshness perception. By integrating visual cue characteristics, psychological processes, and cognitive differences, this study aims to systematically examine how image realism and implied motion in packaging influence perceived food freshness and subsequent purchase intention.

To test the proposed framework, this research employed a sequential three-study design. Fruit juice packaging was selected as the empirical context because freshness is a central quality criterion in this category, while consumers often cannot directly verify product quality prior to purchase (Mehta et al., 2022). In addition, juice packaging frequently relies on fruit imagery and dynamic visual elements, making it a suitable setting for examining image realism and implied motion cues. Study 1 used orange juice packaging, a highly familiar and prototypical juice category, to examine the main effect of image style. Studies 2 and 3 then shifted to kiwi juice packaging to retain category consistency while reducing stimulus-specific associations and repeated-exposure effects. Study 2 tested whether perceived image dynamism mediates the effect of implied motion cues on perceived food freshness, whereas Study 3 further examined whether spatial imagery ability serves as a boundary condition for this mechanism. Together, the three studies provide progressive evidence for the proposed model. The next section presents the conceptual framework and hypotheses.

## 2 Literature review and hypotheses

### 2.1 The impact of packaging cues on perceived food freshness

“Freshness” is cognitively multifaceted and semantically ambiguous. It is treated as a core quality attribute, often linked to “recently manufactured/harvested” (Tran et al., 2024), and has been defined as “closeness to the original state in distance, time, and processing” (Péneau, 2005). Freshness cues are believed to evoke pleasure and reliably guide decisions (Ragaert et al., 2004). Most food and beverage choices are made at the point of sale, with ~90% based on the front of the pack; because in-store tasting is rare, packaging and branding anchor expected flavor (Connolly and Davison, 1996; Simmonds and Spence, 2017).

Prior work has examined multiple packaging cues related to freshness, such as color (Karnal et al., 2016; Schuldt, 2013), spatial layout (Simmonds et al., 2018), logos (Gong et al., 2025), structural design (Fenko et al., 2016; Simmonds and Spence, 2017), health-related claims (Heide and Olsen, 2017), and taste perception (Labbe et al., 2009; Togawa et al., 2019). For example, Heide and Olsen (2017) found that emphasizing the absence of additives, flavorings, or brine in packaging messages can enhance the perceived freshness of fresh foods; circular logos enhance vitality/freshness (Gong et al., 2025); transparent windows signal freshness (Simmonds and Spence, 2017).

Packaging imagery is a vital visual element in juice packaging, and its design features play a critical role in shaping consumer acceptance (Di Cicco et al., 2021), yet graphical elements remain underexamined relative to informational cues. Accordingly, visual imagery is treated as the focal design component. The next section details its effects on food-related perception and derives the research questions.

### 2.2 The impact of realistic images on perceived food freshness

Visual imagery plays a crucial role in conveying information and shaping both cognitive and sensory perception. Compared with verbal information, images are easier to interpret and remember because they require less cognitive effort (Decré and Cloonan, 2019; Krishna et al., 2017). We define depictive realism as the degree to which an image resembles its real-world referent, and distinguish it from technical image quality (e.g., resolution, sharpness, print finish). Both may influence consumer responses, but our theorizing concerns depictive realism rather than technical quality. Depictive realism has been shown to affect consumer responses by reducing psychological distance and narrowing the gap between expectations and experience (Kim et al., 2019). When a depiction closely matches the real object, it is perceived as more realistic; stylized imagery is perceived as less realistic (Ketron et al., 2021). Specifically, photographs are generally perceived as more vivid and realistic than illustrations, and tend to evoke stronger mental imagery and more favorable consumer responses (Hémar-Nicolas et al., 2024). Image realism serves as a major driver of consumer trust and perceived product quality, which in turn influences purchase intention (Orth and Malkewitz, 2008). Aschemann-Witzel et al. (2013) found that the use of realistic versus exaggerated visual representations may lead to different effects on perceived freshness and purchase intention. For example, realistic fruit imagery can evoke perceptions of freshness, quality, and naturalness—attributes that consumers highly value in food products (Becker et al., 2011). Grounded in construal level theory (CLT; Trope and Liberman, 2010), realistic imagery presents the product in concrete, detail-rich form, positioning it as psychologically proximal and experientially accessible. Stylized imagery, by contrast, produces more abstract representations, increasing perceived distance from the actual product state. Because lower psychological distance is associated with more favorable and concrete product evaluations (Ketron et al., 2021; Kim et al., 2019), realistic packaging imagery is expected to strengthen perceived food freshness. We therefore hypothesize that:

*H1*: The presence of realistic imagery (as opposed to stylized imagery) positively influences consumers’ perceived food freshness.

### 2.3 Visual dynamic cues: how implied motion shapes perceived food freshness

Motion is experienced as a perceivable change in position (Radden, 1996), whereas implied motion refers to movement inferred from static visuals through suggestive content or form (e.g., splashing, pouring, bursting) (Yu et al., 2022). Dynamic imagery engages the brain’s capacity to represent movement and to mentally transform or recombine visual input, thereby enabling the perception of dynamism in static images (Paivio and Clark, 1991; Cian et al., 2014). Importantly, implied motion is conceptually distinct from image style. Whereas image style concerns the degree to which an image resembles its real-world referent, implied motion refers to motion-related content inferred from visual elements such as splash trajectories, liquid arcs, or deformation cues. Image style may amplify or clarify such cues, but the proximal driver of perceived image dynamism is the motion-related content itself. To isolate this mechanism, Study 2 manipulated implied-motion content while holding image style constant.

In food contexts, implied motion in imagery has been shown to attract attention and enhance inferences of freshness, naturalness, and quality (López et al., 2024; Xiong et al., 2023; Yu et al., 2022). For example, a splash-like juice image may evoke the impression of a product that has just been squeezed or poured, thereby strengthening freshness-related evaluations. Building on this literature, perceived image dynamism is treated as the proximal psychological mechanism through which implied motion cues inform freshness judgments.

*H2*: Perceived image dynamism mediates the effect of implied motion cues on perceived food freshness.

### 2.4 Individual differences in dynamic perception: the role of spatial imagery ability

Realistic images, by faithfully rendering lighting, texture, and spatial structure, are understood to facilitate contextual construction in viewers, enabling anticipated actions and trajectories and thereby increasing perceived image dynamism (Yim et al., 2021). However, such dynamic perception is not solely determined by the properties of the image; it is also shaped by individual differences among viewers. The visual system, as one of the most critical channels for spatial perception, is widely recognized as a highly complex and efficient information-processing system (Host’ovecký et al., 2019). This incidental encoding capacity may itself vary across individuals as a function of their spatial imagery ability, suggesting that dynamic perception is partly determined by viewer-level cognitive differences.

Spatial imagery ability, as an important component of imagination, refers to the capacity to mentally reconstruct object shapes, structures, and spatial relationships (Kahharov, 2021). This ability is multidimensional and includes shape recognition, pattern visualization, spatial arrangement, and mental rotation, among other skills (Host’ovecký et al., 2019). Prior research suggests that individuals high in spatial imagery ability are better able to infer three-dimensional structure and movement trajectories from two-dimensional images or digital models (Zacks, 2008).

However, in consumer judgment contexts, stronger internal imagery ability may either enhance the interpretation of dynamic cues or reduce reliance on externally provided visual information. Consumers with higher spatial imagery ability may be able to generate richer internal representations of product states, whereas consumers with lower spatial imagery ability may depend more heavily on visible motion-related cues in packaging images. Therefore, spatial imagery ability is expected to moderate the relationship between perceived image dynamism and perceived food freshness. Accordingly, we hypothesize:

*H3*: Spatial imagery ability moderates the positive relationship between perceived image dynamism and perceived food freshness.

**Figure 1:**
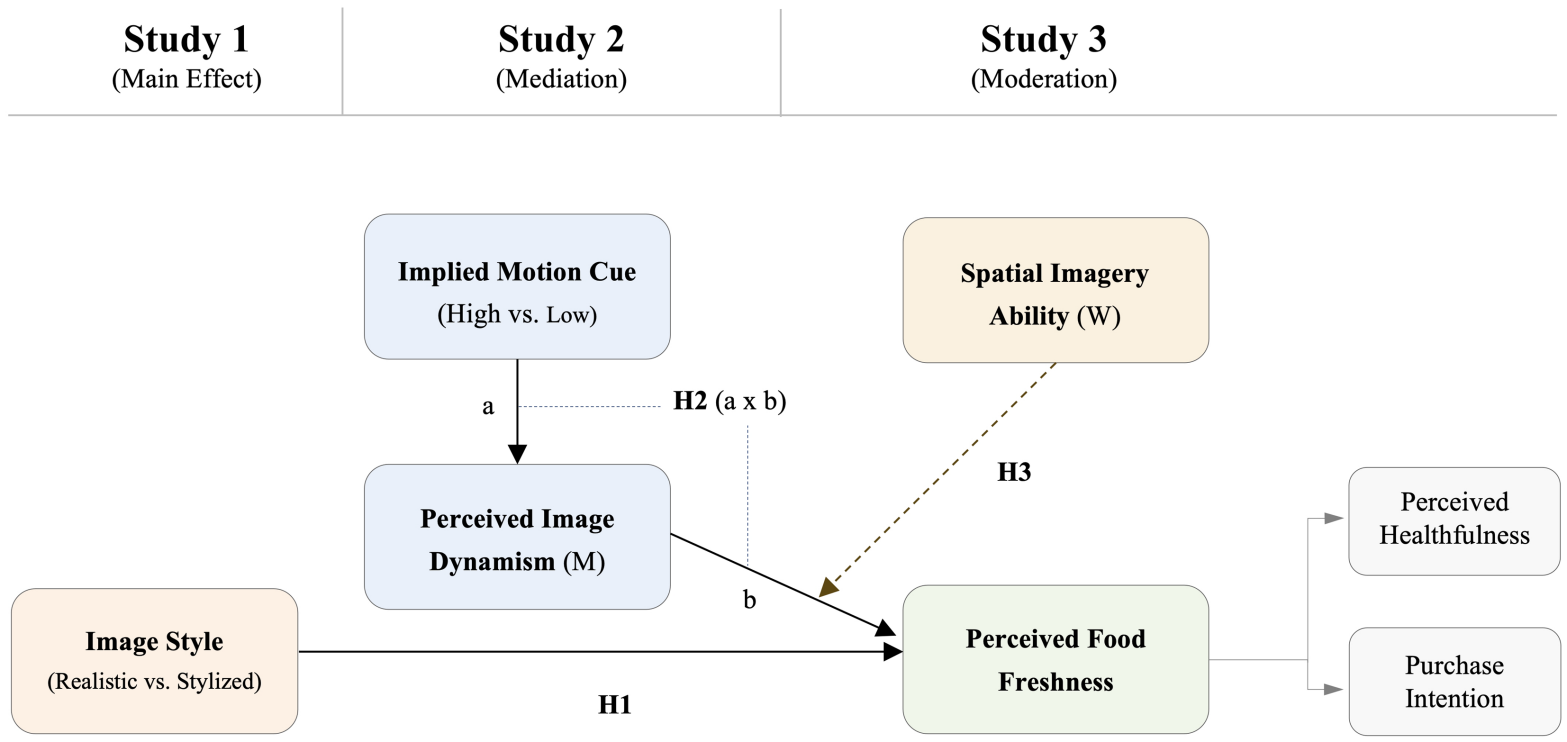
Conceptual model. Source(s): Authors’ work.

Figure 1 illustrates the proposed research framework.

### 2.5 Overview of studies

To test the proposed hypotheses, three studies were conducted in a sequential manner, following the logic of examining main effects, underlying mechanisms, and boundary conditions (Table 1). All studies were administered as online experiments and followed a common procedure. Participants were recruited through a major Chinese online survey platform and randomly assigned to the relevant experimental conditions. All participants reported normal or corrected-to-normal vision. In each study, participants first viewed juice packaging stimuli and then completed the corresponding evaluation measures. Attention-check items and demographic questions were included at the end of the questionnaire, and responses were screened according to predetermined data-cleaning criteria prior to analysis. Because all studies relied on online visual stimuli presented on participants’ own devices, the findings are best interpreted as evidence of how packaging imagery influences subjective judgments under controlled conditions rather than as a full substitute for real-world multisensory packaging experiences.

**Table 1:** Overview of studies.

Study	Study design	Research purpose	Stimulus context	Measurements
1	image style (realistic vs. stylized vs. no-image control)	Main effect	Orange juice packaging	Perceived food freshness; perceived healthfulness; purchase intention
2	implied motion cue (present vs. absent)	Mediation effect	Kiwi juice packaging	perceived image dynamism; Perceived food freshness; perceived healthfulness; purchase intention
3	spatial imagery ability assessed via a 3-item MRT	Moderation effect	Kiwi juice packaging + MRT	Spatial imagery ability

Source(s): Authors’ work.

The research consisted of two independent waves of data collection. Study 1 used an independent sample to examine the main effect of image style on perceived food freshness. Studies 2 and 3 were based on the same data-collection wave, with Study 3 adding a spatial imagery task after completion of the Study 2 experiment in order to test the proposed moderation effect.

Study 1 tested H1 by examining whether image style (realistic vs. stylized) influences perceived food freshness, as well as downstream perceived healthfulness and purchase intention. This manipulation was guided by CLT-based reasoning, with realistic images operationalized as more concrete and product-proximal visual representations than stylized images. Participants viewed juice packaging images presented in different styles and then evaluated the product using 7-point Likert scales.

Study 2 tested H2 by examining whether perceived image dynamism mediates the relationship between implied motion cues and perceived food freshness. Kiwi juice packaging was used as the stimulus to reduce repeated-stimulus effects and fruit-specific associations. The manipulation compared an implied-motion-cue condition with a no-implied-motion condition, operationalized as a splashing image versus a static image. Participants then rated perceived image dynamism and perceived food freshness, and mediation was tested using PROCESS Model 4.

Study 3 examined H3 by testing whether spatial imagery ability, as an individual-difference variable, moderates the effect of perceived image dynamism on freshness judgments. After completing the Study 2 task, participants completed a Mental Rotation Test (Peters et al., 1995), and the moderating effect was tested using PROCESS Model 14. Together, the three studies provide progressive evidence for the proposed model. Detailed descriptions of stimuli and measures are provided in the corresponding sections.

To ensure the validity of inferences and provide robust statistical support, sample size estimates were calculated for each hypothesis using G*Power. For *t*-tests comparing mean differences, a medium effect size (*d* = 0.5) and a statistical power of 0.8 indicated a minimum required sample size of 102 participants. For ANOVA models, assuming a medium effect size (*f* = 0.25), statistical power of 0.8, and a significance level of 5%, the minimum required sample size was 159 participants. The actual sample sizes collected in this research met or exceeded these thresholds, ensuring that all analyses were conducted with sufficient statistical power and inferential validity.

### 2.6 Development of the questionnaire and recruitment of participants

This research employed a structured experimental design to examine how visual cues on juice packaging influence consumers’ perceptions and evaluations of packaged products. Several quality-control procedures were implemented to ensure data quality and reliability. (1) Screening questions were placed at the beginning of the questionnaire to ensure that participants had prior experience purchasing packaged food or beverage products. (2) Attention-check items were embedded in the questionnaire, and participants who failed these checks were excluded. (3) Responses showing invariant answer patterns across all items were removed. (4) Questionnaires completed in less than 60 s were treated as invalid and excluded. These exclusion criteria were applied consistently across all three studies.

The measurement instruments were adapted from validated scales in prior literature (see Table 2) and covered five core constructs: perceived food freshness, perceived healthfulness, purchase intention, perceived image dynamism, and spatial imagery ability.

**Table 2:** Measurement scales and sources.

Construct	Definition	Code and content	Sources	Scale
Perceived food freshness	Consumer’s subjective judgment of food freshness based on visual packaging cues, reflecting heuristic evaluation in low-involvement contexts.	“How fresh does this juice seem?”	Gvili et al. (2015)	7-point
Perceived healthfulness	Subjective inference of product healthiness derived from packaging cues.	“This juice seems healthy.”	Péneau et al. (2009)	7-point
Perceived image dynamism	The extent to which a static package image is perceived as conveying movement, fluidity, or dynamic visual action.	“This image gives me a sense of movement/dynamism.”	López et al. (2024)	7-point
Purchase intention	Consumer’s behavioral intention to purchase or recommend the product.	PI1: “I would buy this product.” PI2: “I would recommend this product to friends.”	Moon et al. (2008)	7-point
Spatial imagery ability	Individual ability to mentally rotate and manipulate visual objects.	MRT: Select the rotated figure matching the target shape	Peters et al. (1995)	Sum of correct responses

Source(s): Authors’ work.

With the exception of spatial imagery ability, all perceptual constructs were measured using seven-point Likert scales (1 = *strongly disagree*, 7 = *strongly agree*). Spatial imagery ability was assessed using an abbreviated version of the Mental Rotation Test (MRT), in which participants selected, from a set of alternatives, the figure that matched a target shape after rotation. Each correct response was scored as 1 point, and the total score—reflecting the sum of correct responses—served as the index of spatial imagery ability, with higher scores indicating greater ability. The questionnaire comprised two sections: the first collected demographic information, and the second assessed participants’ evaluations of the packaging stimuli.

Given that all studies were conducted in an online visual-stimulus context, all scale items were adapted to capture immediate judgments of the packaging image—for example, by replacing product-experience wording with statements such as “This image/this juice appears…”—to improve contextual fit. It should be noted, however, that this approach primarily reflects consumers’ initial responses to online visual stimuli and cannot fully replicate the multisensory packaging experience encountered in real shopping environments, where color rendering, packaging material, tactile interaction, and other sensory cues collectively shape product evaluation.

#### 2.6.1 Recruitment of participants

Across two independent waves of data collection, 711 questionnaires were obtained. After applying the predetermined screening criteria, 592 valid responses were retained for analysis. All participants were recruited through a major Chinese online survey platform and received 15 RMB as compensation. In Study 1, 398 questionnaires were collected, of which 342 were retained as valid responses, resulting in an exclusion rate of 14.1%. Studies 2 and 3 were conducted approximately 2 weeks after Study 1. A total of 313 questionnaires were collected, of which 250 valid responses were retained, corresponding to an exclusion rate of 20.1%. In Study 3, the measure of spatial imagery ability was administered after participants completed the main Study 2 task, in order to further test the proposed boundary condition. Differences in final sample size across studies were mainly due to the predetermined data-cleaning criteria, including failed attention checks, abnormally short completion times, and invalid response patterns.

## 3 Methods and results

### 3.1 Study 1

Study 1 tested H1 by examining whether realistic imagery on juice packaging increases perceived freshness and its downstream effects on healthfulness and purchase intention. A single-factor, between-subjects design was used with three conditions for packaging imagery: realistic, stylized, and control.

#### 3.1.1 Method

**Figure 2:**
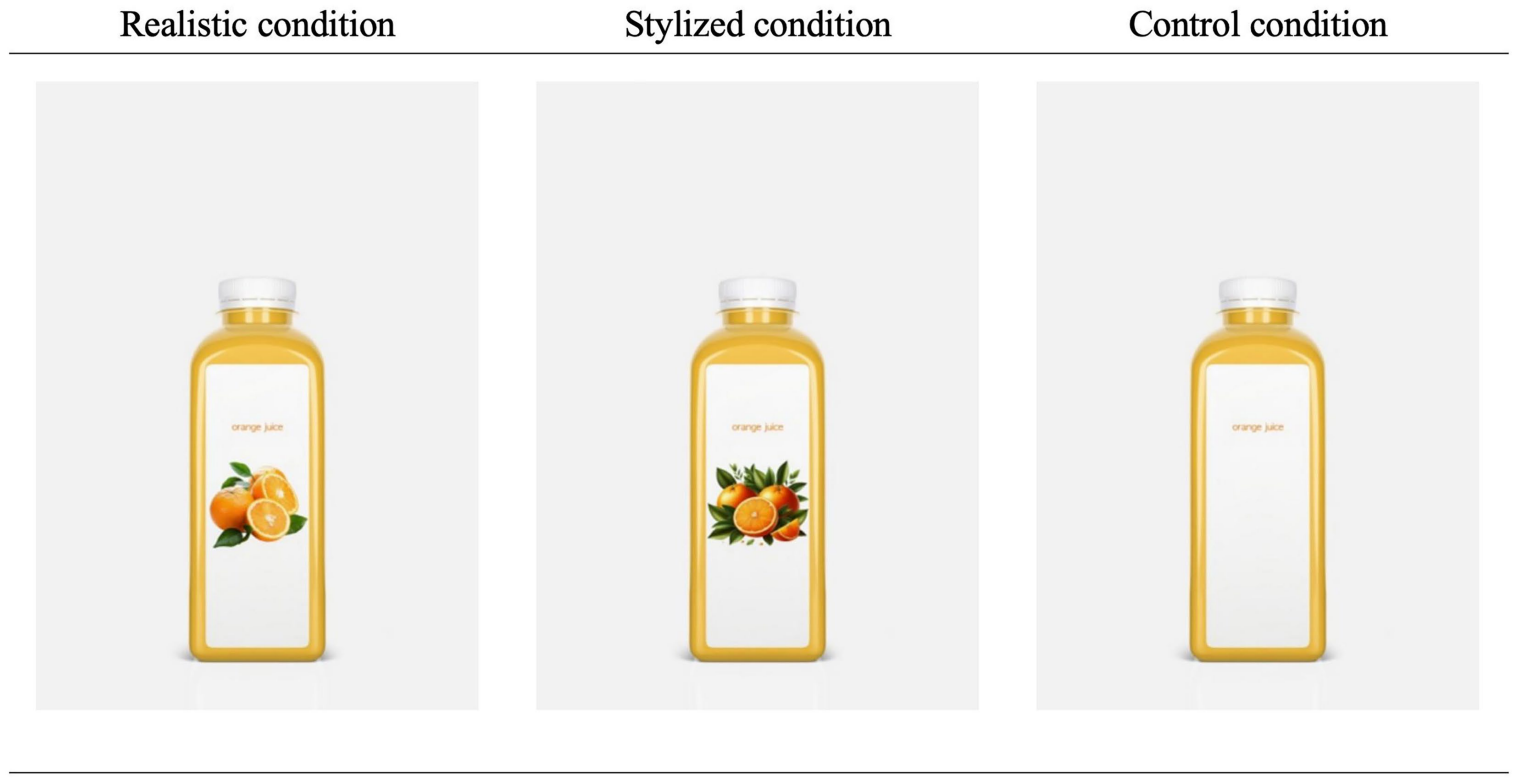
Final designs in Study 1. Source(s): Authors’ work.

Participants were shown a front-facing image of orange juice packaging displaying oranges and the product name “Orange Juice.” The packaging image was presented in one of three conditions—realistic, stylized, or control (see Figure 2). Visual and typographic elements were created in Adobe Photoshop CC 2020. Participants were recruited through an online survey platform and were required to have normal or corrected-to-normal vision to ensure proper perception of the visual stimuli. After providing informed consent, participants were randomly assigned to one of the three experimental conditions and completed the questionnaire.

Perceived food freshness was measured with a single-item 7-point scale: “How fresh does the juice seem?” (1 = Not fresh at all, 7 = Very fresh) (Gvili et al., 2015). Perceived healthfulness was assessed using a single-item seven-point scale adapted from prior research (Péneau et al., 2009). Purchase intention was measured using two items adapted from prior research (Moon et al., 2008), and the scale showed good internal consistency (Cronbach’s *α* = 0.85). Demographic information was also collected.

#### 3.1.2 Participants

A total of 398 consumers in China were recruited to participate in the study in exchange for a 15 RMB incentive. Only those who passed the attention check were retained for analysis. Descriptive statistics for the final sample in Study 1 (*n* = 342) are presented in Table 3.

**Table 3:** Demographics for Study 1, Study 2 and Study 3.

Item	Category	Study 1	Study 2
			Study 3
*N*		*342*	*250*
Gender	Male	38.89%	40.8%
	Female	61.11%	59.2%
Age	Under 18	1.46%	1.6%
	18–24	55.26%	64.8%
	25–34	29.82%	24.4%
	35–44	10.23%	6.4%
	45–54	2.05%	1.2%
	55 and above	1.07%	1.6%
Educational level	Less than bachelor’s degree	25.14%	30.4%
	Bachelor’s degree	61.11%	56.4%
	Master’s degree	13.74%	13.2%
Employment status	Students	54.68%	65.2%
	Employee	30.41%	19.6%
	Freelance	8.48%	9.2%
	Self-employed	4.39%	4.0%
	Retired	1.17%	1.6%
	Others	0.88%	0.4%
Revenue range (RMB)	Between 1 and 3,000	42.11%	50.8%
	Between 3,001 and 5,000	21.64%	22.8%
	Between 5,001 and 7,000	20.18%	18%
	Between 7,001 and 9,000	9.36%	4.8%
	Above 9,000	6.73%	3.6%

Source(s): Authors’ work.

The sample consisted of 61.11% female participants. In terms of age, 55.26% were between 18–24 years old, 29.82% between 25–34, and 10.23% between 35–44. Among respondents, 61.11% held a bachelor’s degree, and at least 13.74% had obtained a master’s degree. Regarding income, 42.11% reported earning less than 3,000 RMB per month, and 21.64% reported earning between 3,001 and 5,000 RMB.

#### 3.1.3 Manipulation check

To assess the effectiveness of the manipulation of image realism, participants were asked to rate the perceived realism of the fruit image they viewed on a 7-point scale (1 = Not at all realistic, 7 = Very realistic) (Hémar-Nicolas et al., 2024). An ANOVA confirmed a significant difference in perceived realism ratings across the three image types, *F*(2, 339) = 1170.50, *p* < 0.001, η^2^ = 0.874. *Post hoc* comparisons revealed that the realism ratings in the realistic photo condition (*M* = 6.33, SD = 0.533) received significantly higher realism ratings than the stylized condition (*M* = 4.66, SD = 0.867) and the no-image control condition (*M* = 1.50, SD = 0.837); moreover, the stylized condition also significantly exceeded the control condition. All pairwise *p* < 0.001. These results indicate that the manipulation of image realism was successful, as participants were able to distinguish between different levels of image realism.

#### 3.1.4 Results and discussion

Study 1 treated perceived food freshness as the focal outcome variable. In addition, perceived healthfulness and purchase intention were included as downstream outcomes to examine whether the effect of image style extends to broader product evaluations and behavioral intentions.

Perceived food freshness. A significant main effect of image type on perceived freshness was observed, *F*(2, 339) = 119.529, *p* < 0.001, ηp^2^ = 0.414, supporting H1. Post hoc tests indicated higher freshness in the realistic condition (*M* = 5.64, SD = 1.345) than in the stylized (*M* = 4.57, SD = 1.358, *p* < 0.001) and control conditions (*M* = 2.73, SD = 1.574, *p* < 0.001). The lowest freshness occurred in the no-image control, underscoring the communicative value of visual elements and aligning with prior work (Gvili et al., 2015).

Perceived healthfulness. Image type also affected perceived healthfulness, *F*(2, 339) = 70.923, *p* < 0.001, ηp^2^ = 0.295. Ratings were highest for realistic imagery (*M* = 5.45, SD = 1.418), followed by stylized (*M* = 4.53, SD = 1.435) and control (*M* = 3.03, SD = 1.753).

Purchase intention. A main effect of image type on purchase intention was found, *F*(2, 339) = 33.975, p < 0.001, ηp^2^ = 0.167. Intentions were higher with realistic imagery (*M* = 4.87, SD = 1.403) than with stylized (*M* = 4.24, SD = 1.416) or control (*M* = 3.28, SD = 1.536); all pairwise differences were significant (*p* < 0.001). Thus, perceived image realism elevated purchase intention, and even stylized imagery increased appeal relative to no image.

Collectively, Study 1 demonstrates that realistic package imagery generates stronger perceived food freshness than stylized or absent imagery, and that this advantage further extends to perceived healthfulness and purchase intention (see Table 4).

**Table 4:** Study 1 results: effects of image style on consumer perceptions.

Measures	Realistic condition *N* = 119	Stylized condition *N* = 115	Control condition *N* = 108	*F*	η_p_^2^
	Mean (SD)	Mean (SD)	Mean (SD)		
Perceived food freshness	5.64 (1.345)	4.57 (1.358)	2.73 (1.574)	119.529***	0.414
Perceived healthfulness	5.45 (1.418)	4.53 (1.435)	3.03 (1.753)	70.923***	0.295
Purchase intention	4.87 (1.403)	4.19 (1.506)	3.28 (1.536)	33.975***	0.167

***p < 0.001.

Source(s): Authors’ work.

### 3.2 Study 2

Study 2 was designed to further test H2 by examining whether perceived image dynamism mediates the effect of implied motion cues on perceived food freshness. To clarify this mediating pathway and reduce potential confounds arising from stimulus repetition or fruit-specific associations, kiwi juice packaging was used as the stimulus. Image style was held constant in a realistic style, whereas implied-motion content was manipulated by comparing a splashing image with a static image. This design allowed us to isolate the psychological mechanism through which perceived image dynamism contributes to freshness judgments.

#### 3.2.1 Method

A between-subjects design was employed with two image conditions: an implied-motion-cue condition and a no-implied-motion condition. The independent variable was image condition, the mediator was perceived image dynamism, and the dependent variable was perceived food freshness.

**Figure 3:**
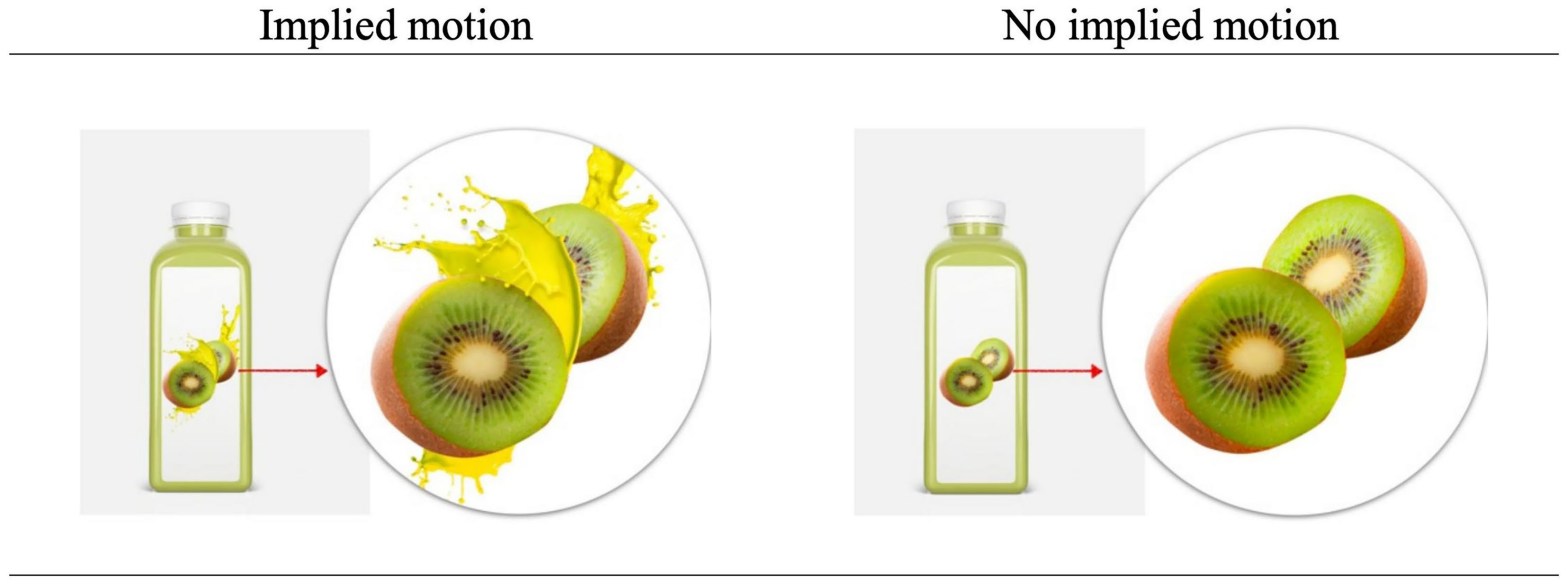
Final designs in Study 2. Source(s): Authors’ work.

Participants viewed a front-facing kiwi juice package. Two package variants were created (see Figure 3). Both versions displayed sliced kiwi fruit, but only one included a splashing-juice effect to convey implied motion. The other version showed the fruit without motion-related elements and served as the static condition. All other packaging elements, including product type, layout, color scheme, and textual information, were held constant.

A total of 313 Chinese participants were recruited through an online survey platform and received 15 RMB as compensation. After attention checks and data screening, 250 valid responses were retained for analysis. Among the final sample, 59.2% were female, and most participants were between 18 and 34 years old. Detailed demographic information is presented in Table 3.

Participants completed an online questionnaire after viewing the assigned package image. Perceived food freshness and perceived image dynamism were measured using seven-point scales. Purchase intention was measured with two items and showed good internal consistency (Cronbach’s *α* = 0.92). The data were then used to test whether perceived image dynamism mediates the effect of implied motion cues on perceived food freshness.

#### 3.2.2 Manipulation check

To assess whether the implied motion manipulation was successful, an independent-samples t-test was conducted on perceived image dynamism. Results indicated that participants in the implied-motion-cue condition reported significantly higher perceived image dynamism than those in the no-implied-motion condition (*M* = 5.48, *M* = 4.35; *t*(244.03) = 5.827, *p* < 0.001, Cohen’s d = 1.531), confirming that participants clearly perceived different levels of implied motion across the two image conditions.

#### 3.2.3 Results and discussion

To test whether perceived image dynamism mediates the effect of implied motion cues on perceived food freshness, independent-samples t tests were first conducted.

Perceived food freshness. The implied-motion group reported higher freshness than the no implied motion group (*M* = 5.41, SD = 1.198; *M* = 4.82, SD = 1.331; *t*(243) = 3.677, p < 0.001, Cohen’s d = 1.265). Moreover, participants in the implied motion group also reported significantly higher perceived motion scores (*M* = 5.48, SD = 1.171; *M* = 4.35, SD = 1.294; *t*(244.03) = 5.827, *p* < 0.001, Cohen’s d = 1.531). These results indicate that implied motion cues not only enhanced consumers’ perception of image dynamism but also increased their subjective evaluation of product freshness.

Perceived healthfulness did not differ significantly between the two conditions, *t*(245.75) = 0.621, *p* = 0.535. We then conducted a mediation analysis using PROCESS Model 4 with 5,000 bootstrapping resamples, as proposed by Hayes and Preacher (2014). Perceived image dynamism was specified as the mediator between implied motion cues and perceived food freshness. The results indicated a significant indirect effect of implied motion cues on perceived food freshness through perceived image dynamism [indirect effect = 0.417, SE = 0.098, 95% CI (0.247, 0.629)]. Therefore, H2 was supported.

### 3.3 Study 3

Study 3 was designed to examine the boundary conditions of the proposed effects by testing the moderating role of spatial imagery ability. Dynamic visual cues on packaging often require consumers to mentally simulate motion from a static image. Accordingly, individual differences in the ability to process and transform spatial information may influence the extent to which consumers rely on such cues when forming freshness judgments. Building on the theoretical rationale and hypothesis developed in Section 2.4, Study 3 empirically tested whether spatial imagery ability moderates the indirect effect of image condition on perceived food freshness via perceived image dynamism.

#### 3.3.1 Method

Study 3 was conducted using the same experimental stimuli and valid sample as Study 2. Participants were randomly assigned to one of two image conditions (implied motion vs. no implied motion) using kiwi juice packaging stimuli. After completing the Study 2 evaluations, participants additionally completed a Mental Rotation Test to assess spatial imagery ability.

In this moderated mediation model, the independent variable was image condition, perceived image dynamism served as the mediator, perceived food freshness was the dependent variable, and spatial imagery ability was entered as a continuous moderator.

#### 3.3.2 Participants

Study 3 drew on the same sample as Study 2 (*N* = 250; 59.2% female; detailed demographics reported in Section 3.2.1). In addition to the Study 2 measures, participants completed a spatial imagery ability assessment.

Spatial imagery ability was assessed with three items adapted from the Mental Rotation Test (Peters et al., 1995), a widely used measure of the capacity to mentally rotate three-dimensional shapes. The MRT was originally developed by Vandenberg and Kuse (1978) and revised by Peters et al. (1995) to improve its psychometric properties. Within a limited time, participants judged whether several figures were rotated versions of a target figure. The score was the total number of correct responses across the three items, with higher scores indicating greater spatial imagery ability.

#### 3.3.3 Results and discussion

To test the proposed moderated mediation model (H3), conditional process analysis was employed using PROCESS Model 14.

Path estimates. The manipulated image condition positively predicted perceived image dynamism (*B* = 1.144, SE = 0.194, *t* = 5.895, *p* < 0.001). Perceived image dynamism positively predicted perceived food freshness (*B* = 0.535, SE = 0.090, *t* = 5.890, *p* < 0.001). A significant interaction emerged between perceived image dynamism and spatial imagery ability (*B* = −0.102, SE = 0.048, *t* = −2.101, *p* = 0.036), indicating moderation. Conditional effect analysis further revealed that when spatial imagery ability was low (*w* = 1.00), the effect of perceived image dynamism on perceived food freshness was strong and significant [*B* = 0.433, SE = 0.054, 95% CI (0.326, 0.539)]; at high spatial imagery ability (*w* = 2.00), the effect remained significant but weaker [*B* = 0.330, SE = 0.048, 95% CI (0.233, 0.426)].

These findings suggest that individuals with lower spatial imagery ability tend to rely more heavily on motion cues within the image when making freshness judgments. The index of moderated mediation was −0.117 [95% CI (−0.242, −0.006)], excluding zero and confirming that spatial imagery ability significantly moderated the indirect effect of image condition on perceived food freshness via perceived image dynamism. Together, these results support H3 and suggest that the effectiveness of dynamic packaging cues in signaling freshness is contingent on consumers’ spatial cognitive resources.

## 4 Discussion

This study examined how juice packaging imagery shapes consumers’ perceived food freshness by developing and testing a framework that integrates a main visual effect, an underlying psychological mechanism, and an individual-difference boundary condition. Rather than treating image realism, implied motion, and spatial imagery ability as separate issues, the present research connects them within a single explanatory logic, offering a more complete account of how packaging visual cues translate into freshness-related consumer judgments.

Theoretically, this research extends the literature on packaging cues and consumer perception in several ways. Prior studies have examined how packaging elements such as textual labels, color, and structural features shape consumer judgments of product attributes (Heide and Olsen, 2017; Karnal et al., 2016). However, relatively less attention has been paid to the representational form of package imagery itself. The present findings show that image style is not merely an aesthetic choice, but an informational cue that influences how consumers infer product state. Compared with stylized imagery, realistic imagery appears to reduce the inferential distance between the package image and the actual product, making the image more likely to be interpreted as a credible representation of freshness, naturalness, and quality. Consistent with the CLT-based reasoning advanced in Section 2.2, realistic imagery renders the product in concrete, low-level detail, thereby fostering a sense of psychological proximity that stylized imagery is less able to provide (Trope and Liberman, 2010). This finding supports prior work showing that image realism can reduce psychological distance and improve product evaluation (Ketron et al., 2021), while extending this logic to perceived food freshness, a category-relevant attribute in juice packaging.

This study also clarifies the psychological mechanism through which implied motion cues influence freshness judgments. Prior research has shown that implied motion can affect perceived healthfulness, expected taste, and product attractiveness (Gong et al., 2025; Yu et al., 2022). However, these studies have often focused on outcome effects rather than explaining why such visual cues shape freshness-related inferences. The present results suggest that consumers do not simply interpret splashing, pouring, or flowing elements as decorative features. Instead, these cues increase perceived image dynamism, making the product appear more active, fluid, and temporally immediate. This perceived dynamism is then translated into a stronger judgment of product freshness. In this sense, perceived image dynamism specifies a measurable psychological pathway through which static packaging images can create a sense of “freshly prepared,” “just poured,” or “currently active” product states.

The identification of perceived image dynamism as a mediating mechanism carries a broader theoretical implication. These findings further suggest that consumers are not passive recipients of visual freshness signals but actively construct product-state inferences from visual cues (Elder and Krishna, 2022). For juice products, this process is particularly relevant because freshness is closely associated with immediacy, naturalness, and sensory vitality. The contribution of this study is not only to show that dynamic imagery is effective, but also to explain why consumers may interpret dynamic visual content as a freshness-related signal in juice packaging.

A further contribution lies in identifying spatial imagery ability as a boundary condition. Cognitive research often emphasizes that individuals with higher spatial ability are better at processing spatial relations, motion trajectories, and internal representations (Zacks, 2008). One might therefore expect these consumers to be more responsive to dynamic imagery. However, the present findings show a more nuanced pattern. In the context of juice packaging, the positive effect of perceived image dynamism on perceived food freshness was stronger among consumers with lower spatial imagery ability. A plausible explanation is that consumers with higher spatial imagery ability can generate richer internal representations of product states even when external motion cues are less explicit. As a result, they may depend less on visible dynamic cues. By contrast, consumers with lower spatial imagery ability may have fewer internal resources for mentally simulating product movement and therefore rely more heavily on explicit motion-related cues embedded in the package image. This finding suggests that cognitive ability does not always amplify the persuasive effect of visual cues; it may also reduce consumers’ dependence on external cues by enabling internal simulation (Pearson, 2019). This pattern challenges the common assumption that higher cognitive ability uniformly amplifies the persuasive impact of visual cues. Instead, the present findings suggest that cognitive ability may operate as a compensatory resource: when internal simulation capacity is sufficient, external cues become less necessary. This insight extends current understanding of individual differences in visual persuasion beyond a simple “more ability = stronger response” model.

From a managerial perspective, the findings offer several implications for juice packaging design and brand communication. First, brands that aim to communicate freshness, naturalness, and product quality may benefit from using realistic fruit or product imagery. Realistic imagery can make the product appear closer to its actual consumption state, thereby strengthening perceived freshness and downstream purchase intention (Becker et al., 2011; Ketron et al., 2021). From a CLT perspective, this reflects the role of packaging imagery in managing consumers’ psychological distance from the product — a dimension that practitioners can actively calibrate through the choice between realistic and stylized visual representations. Second, designers may strategically incorporate implied motion elements—such as liquid splashes, dynamic pours, or effervescent bursts—to enhance perceived image dynamism. These cues may be especially useful in retail or online settings where consumers cannot directly evaluate smell, taste, or texture before purchase. In such situations, dynamic visual cues can partially compensate for limited sensory access by making the product appear more vivid and immediate (Yu et al., 2022; Fenko et al., 2010). Third, motion cues should not be treated as universally beneficial or simply maximized. Their effectiveness may depend on the target consumer’s cognitive processing characteristics and the communication context. For consumers with lower spatial imagery ability, stronger and more explicit dynamic cues may be particularly effective in communicating freshness. For consumers with higher spatial imagery ability, more restrained motion cues may suffice, as these consumers can generate richer internal representations independently; overly complex dynamic elements may therefore add unnecessary visual complexity without proportional gains in perceived freshness.

Overall, this study demonstrates that juice packaging imagery is not merely decorative but functions as a meaningful informational cue through which consumers infer product freshness. By integrating image realism, implied motion, perceived image dynamism, and spatial imagery ability into a unified framework, this research advances understanding of how visual packaging design influences consumer perception. More broadly, the findings suggest that the effectiveness of visual cues in food marketing is shaped not only by the properties of the stimulus but also by the cognitive resources consumers bring to the evaluation process. This perspective encourages future researchers and practitioners to move beyond a stimulus-centric view of packaging design and to consider the consumer’s cognitive context as an integral part of visual communication effectiveness.

## 5 Limitations and directions for future research

This research has several limitations that suggest directions for future work. First, the studies used controlled, imagery-based online experiments. Although this approach strengthened internal validity, it may not fully capture the multisensory complexity of real shopping environments. Because participants completed the studies using their own devices, differences in screen color, brightness, and resolution could not be fully controlled, potentially influencing perceptions of image realism, implied motion cues, and freshness judgments. More fundamentally, the reliance on purely visual stimuli means that the theoretical framework developed here—linking visual cues to freshness inferences via mental simulation—has not yet been tested in contexts where multisensory integration occurs. Future research should consider more ecologically valid settings, such as virtual reality simulations, laboratory-based shelf displays, or field experiments, to examine how visual packaging cues interact with other sensory inputs, including smell, touch, and sound, in shaping freshness judgments and purchase decisions.

Second, the empirical scope of this research was limited to juice packaging, a category in which freshness is a central evaluative criterion and product quality cannot be directly verified prior to purchase. This boundary condition is theoretically meaningful: the proposed effects may be strongest in categories where consumers must rely on visual inference rather than direct sensory access. Accordingly, the present findings should not be generalized directly to highly processed foods, shelf-stable products, or categories where freshness is not a primary purchase consideration. Future studies could extend the framework to other product categories to test whether the mechanisms identified here—particularly the mediating role of perceived image dynamism—remain operative when freshness is less central to consumer evaluation.

Third, this study focused exclusively on visual packaging cues, specifically image realism and implied motion, without examining how they interact with other elements of the packaging system. Theoretically, packaging operates as a multisemiotic system in which visual, textual, structural, and material cues may jointly or competitively shape consumer perception (Piqueras-Fiszman and Spence, 2015). The present framework isolates visual cues for analytical clarity, but this comes at the cost of ecological completeness. Future research could investigate how dynamic visual cues interact with verbal freshness claims, packaging structure, or material texture, as such interactions may amplify, attenuate, or redirect the freshness-signaling effects identified in this study.

Fourth, the study relied mainly on self-reported measures. Perceived food freshness, perceived image dynamism, perceived healthfulness, and purchase intention were measured using rating scales, while spatial imagery ability was assessed with a shortened three-item Mental Rotation Test. Future work could combine self-reports with behavioral measures, more complete cognitive tests, eye-tracking data, or reaction-time indicators to better capture the psychological processes underlying visual cue use.

Finally, this research was conducted exclusively within a Chinese consumer context using convenience sampling, which limits the generalizability of the findings. Cultural differences in visual processing styles, freshness norms, and packaging aesthetics may moderate the effects observed here. For instance, prior research suggests that consumers from different cultural backgrounds vary in their sensitivity to visual realism and motion-related cues (Cian et al., 2014). Future research should test the robustness of the proposed framework across different cultural settings and more representative consumer samples to establish its boundary conditions and cross-cultural generalizability.

## Data Availability

The raw data supporting the conclusions of this article will be made available by the authors, without undue reservation.
